# Safety monitoring of ROTAVAC vaccine and etiological investigation of intussusception in India: study protocol

**DOI:** 10.1186/s12889-018-5809-7

**Published:** 2018-07-20

**Authors:** Samarasimha Reddy, Nayana P. Nair, Sidhartha Giri, Venkata Raghava Mohan, Jacqueline E. Tate, Umesh D. Parashar, Mohan D. Gupte, Rashmi Arora, Gagandeep Kang, Indian Intussusception Surveillance Network, Sowmiya V. Senthamizh, G. S. Rama Prasad, Suhasini Mekala, Bhaskar Reddy, Goru Krishna Babu, Padmalatha Pamu, Rajendra Prasad Gorthi, Vittal Mohan, Manohar B, Subal Pradhan, Hiranya Mohanty, Mrutunjay Dash, J. Bikrant Kumar Prusty, Nirmal Kumar Mohakud, Subrat Mohanty, Rajib Ray, Prasantajyoti Mohanty, Geetha Gathwala, K. N. Rattan, Suraj Chawla, M. S. Yamini, Madhu Gupta, Surjit Singh, R. K. Gupta, Arun Gupta, Suresh Goyal, Deendayal Sharma, Pramod Sharma, Sunil Kothari, Gopinath, Balasubramaniyam S, Jai Durai Raj, Girish Kumar CP, Sridevi A. Naraayan, Muthu Kumar J, Kulandaivel S, Vaijayanthi G, Hemanth Kumar B, Raghul Maniam, Rajamani Gurusamy, Kumaravel S, Ashwitha Shenoy, Brijesh Lahoti, Sharad Thora, Pawan Ghanghoriya, Vikesh Agarawal, Koshy George, Sam Joel, Jayanta Goswami, Ashish Wakhlu, Vineeta Gupta, S. P. Sharma, Mahima Mithal

**Affiliations:** 1 The Wellcome Trust Research Laboratory, Division of Gastrointestinal Sciences, Christian Medical College, Vellore, Tamil Nadu India; 2 Department of Community Health, Christian Medical College, Vellore, Tamil Nadu India; 3 Centers for Disease Control and Prevention, Atlanta, GA USA; 4 Indian Council of Medical Research, New Delhi, India; 5 Translational Health Science and Technology Institute, Faridabad, India

**Keywords:** ROTAVAC, Rotavirus vaccine, Intussusception, India, Self-controlled case series methods, Infectious etiologies

## Abstract

**Background:**

ROTAVAC, an indigenous rotavirus vaccine, was introduced in the universal immunization program of India in four states in 2016 and expanded to five more states in 2017. The clinical trial on efficacy of ROTAVAC did not detect an increased risk of intussusception, but the trial was not large enough to detect a small risk. This protocol paper describes the establishment and implementation of a surveillance system to monitor the safety of rotavirus vaccine and investigate the potential infectious etiologies of intussusception.

**Methods:**

This is a multi-centric hospital-based active surveillance being conducted at 28 hospitals in nine states of India. Data gathered from surveillance will be used to assess the risk of intussusception after ROTAVAC administration and to determine the infectious etiologies of intussusception. For safety assessment of ROTAVAC vaccine, children aged less than two years with intussusception admitted at the sentinel hospitals are enrolled into surveillance, a case report form completed, and a copy of the vaccination card obtained. The risk of intussusception following rotavirus vaccination will be assessed using a self-controlled case-series design. The investigation for potential infectious etiologies of intussusception is through a matched case-control design. Children enrolled for the safety assessment serve as cases and for each case, an age, gender and location matched control is enrolled within 30 days of case enrollment. Stool specimens are obtained from cases and controls. All forms and specimens are sent to the referral laboratory for data entry, analysis, multiplexed molecular testing, and storage.

**Discussion:**

Anticipated public health benefits of this surveillance include the generation of information useful to national government on safety of vaccine and to make future decisions on vaccine use through risk-benefit analysis. Investigating infectious agents may help to determine the potential infectious etiologies of intussusception.

**Electronic supplementary material:**

The online version of this article (10.1186/s12889-018-5809-7) contains supplementary material, which is available to authorized users.

## Background

Rotavirus (RV) is the commonest cause of severe gastroenteritis worldwide, accounting for 215,000 deaths annually among children under five years of age [1]. In India, based on the 2011 birth cohort, RV gastroenteritis causes an estimated 11.37 million illness episodes, 3.27 million outpatient visits and 872,000 inpatient admissions each year resulting in direct costs of USD 172.8 million each year [2]. RV caused approximately 39% of gastroenteritis hospitalizations and 78,000 deaths among Indian children under five years of age [2]. The World Health Organization (WHO) recommends the introduction of rotavirus vaccines in all countries and particularly, in countries with high child mortality due to gastroenteritis [3]. ROTAVAC (Bharat Biotech), an indigenously developed monovalent, live attenuated oral rotavirus vaccine containing the 116E strain (G9P[11]) [4], is being introduced in the Universal Immunization Program (UIP) of India in a phased manner with initial introduction in four states in 2016 and five additional states in 2017, with others states to follow [5, 6].

A key issue for rotavirus vaccines is safety, especially with regard to intussusception, a severe but uncommon intestinal blockage [7]. An earlier rotavirus vaccine based on a different (rhesus) strain and introduced in the United States (US) in the late 1990s [8, 9] was associated with an increased risk of intussusception and hence withdrawn from the US market [10, 11]. A low-level risk of 1–6 excess cases per 100,000 vaccinated children has been identified with both monovalent Rotarix (RV1, GlaxoSmithKline) and pentavalent Rotateq (RV5, Merck) vaccines in several high- and middle-income countries [12, 13]. The clinical trial on the efficacy of ROTAVAC vaccine did not detect an increased risk of intussusception among vaccinated infants, however, the trial was not large enough to detect a small risk [4]. Additionally, while the risk to benefit ratio of rotavirus vaccines are in favour of the vaccine [8, 14], this rare adverse effect has been highly publicized, and it is important to generate data on intussusception [15, 16]. The WHO recommends data collection on intussusception for rotavirus vaccines using sentinel hospitals [17]. Therefore, establishing a network of health care facilities that recognize and manage cases of paediatric intussusception will help in evaluating the association between intussusception and ROTAVAC vaccination after introduction of the vaccine into the universal immunization programme (UIP) of India.

Although the etiology of intussusception in infancy and early childhood is not very clear, infections are commonly hypothesized to be associated with intussusception in this age group [18, 19]. Certain viruses such as adenoviruses have been found at lead points in intussusception [18, 19]. Some clinical studies have reported high prevalence rates of certain viruses in stool samples from intussusception cases [20]. The evidence for the association of enteric pathogens with intussusception is inconclusive, as most of these studies on infectious etiology have failed to do a comparative analysis between pathogens found in intussusception stool samples versus those found in samples from age-matched healthy controls. Among the very few studies which have tried to evaluate multiple infectious etiologies using a case-control approach, a study conducted in Vietnam and Australia found a strong association with adenovirus, but not with other pathogens [21]. Similar data on infectious etiology of intussusception from Indian settings is currently lacking.

This protocol paper describes the methods for establishment and implementation of an intussusception surveillance system in India to monitor the safety of rotavirus vaccine following its introduction into the UIP and to investigate the potential infectious etiologies of intussusception.

## Methods

### Objectives

The objectives of intussusception surveillance are:

- *Primary objective*

To assess the risk of intussusception following ROTAVAC administration using the self-controlled case-series method.

- *Secondary objective*

To describe potential infectious etiologies of intussusception by testing for a wide range of enteric pathogens in stool samples of intussusception cases and matched controls using sensitive molecular methods.

### Project management

The project is managed by the Christian Medical College (CMC), Vellore, and the Centers for Disease Control and Prevention (CDC), Atlanta, USA in collaboration with the Translational Health Science and Technology Institute (THSTI), Faridabad and the Indian Council of Medical Research (ICMR). CMC, Vellore is responsible for all administrative arrangements, while monitoring the surveillance is jointly done by CMC, THSTI and ICMR.

### Site selection and surveillance initiation

The criteria for sentinel hospitals to participate in surveillance include the ability to diagnose and manage cases of intussusception (availability of pediatric surgeon, radiologist, equipment, and facilities to manage intussusception). We selected large tertiary care hospitals in states introducing the vaccine as part of the UIP, and requested their participation. A meeting was organised for site representatives of potential sentinel hospitals. Representative from each site were requested to provide details on the facilities available in their hospitals for managing intussusception cases and retrospective data on intussusception admissions among children aged less than two years for a period of one year (Additional file 1: Table S1). Sites with appropriate expertise and infrastructure to manage intussusception cases were selected to participate in the surveillance program. Active surveillance was initiated at 28 sentinel hospitals including two hospitals from states/union territories without rotavirus vaccine as part of the UIP, but which serve as referral centres admitting cases of intussuception from states with rotavirus vaccination (Table 1). A memorandum of understanding (MoU) was signed between each sentinel hospital and CMC, Vellore. During surveillance initiation, training sessions were conducted to the surveillance staff at each hospital, which includes one or more pediatric surgeons, radiologists, pediatricians/community health physicians, and a field research assistant.

**Table 1:** Sentinel hospitals in Intussusception surveillance

S. No.	Surveillance Network Centers	Location	State
1	Kurnool Medical College	Kurnool	Andhra Pradesh
2	Government General Hospital	Kakinada	Andhra Pradesh
3	King George Hospital	Vishakhapatnam	Andhra Pradesh
4	Sri Venkateswara Medical College	Tirupati	Andhra Pradesh
5	Sardar Vallabhai Patel Post Graduate Institute of Paediatrics	Cuttack	Odisha
6	Kalinga Institute of Medical Sciences	Bhubaneswar	Odisha
7	Institute of Medical Sciences and SUM Hospital	Bhubaneswar	Odisha
8	Hi-Tech Hospital	Bhubaneswar	Odisha
9	Pandit Bhagwat Dayal Sharma Post Graduate Institute of Medical Sciences	Rohtak	Haryana
10	Shaheed Hasan Khan Mewati Government Medical College	Mewat	Haryana
11	Post Graduate Institute of Medical Education and Research	Chandigarh	Chandigarh
12	Sawai Man Singh Medical college	Jaipur	Rajasthan
13	Rabindranath Tagore medical college	Udaipur	Rajasthan
14	Dr. Sampurnanand Medical college	Jodhpur	Rajasthan
15	Christian Medical College	Vellore	Tamil Nadu
16	Government Vellore Medical college	Vellore	Tamil Nadu
17	Institute of Child health	Chennai	Tamil Nadu
18	Kanchi Kama Koti Child Trust hospital	Chennai	Tamil Nadu
19	Government Medical College	Madurai	Tamil Nadu
20	Government Medical College	Coimbatore	Tamil Nadu
21	Jawaharlal Nehru Institute of Post-graduate Medical Education & Research (JIPMER)	Puducherry	Puducherry
22	Mahatma Gandhi Memorial Medical College	Indore	Madhya Pradesh
23	NSCB Medical college	Jabalpur	Madhya Pradesh
24	King George Medical College	Lucknow	Uttar Pradesh
25	Institute of Medical Sciences, Banaras Hindu University	Varanasi	Uttar Pradesh
26	BRD Medical College	Gorakhpur	Uttar Pradesh
27	Baptist Christian Hospital	Tezpur	Assam
28	Government Medical college	Guwahati	Assam

### Setting and design

**Fig. 1:**
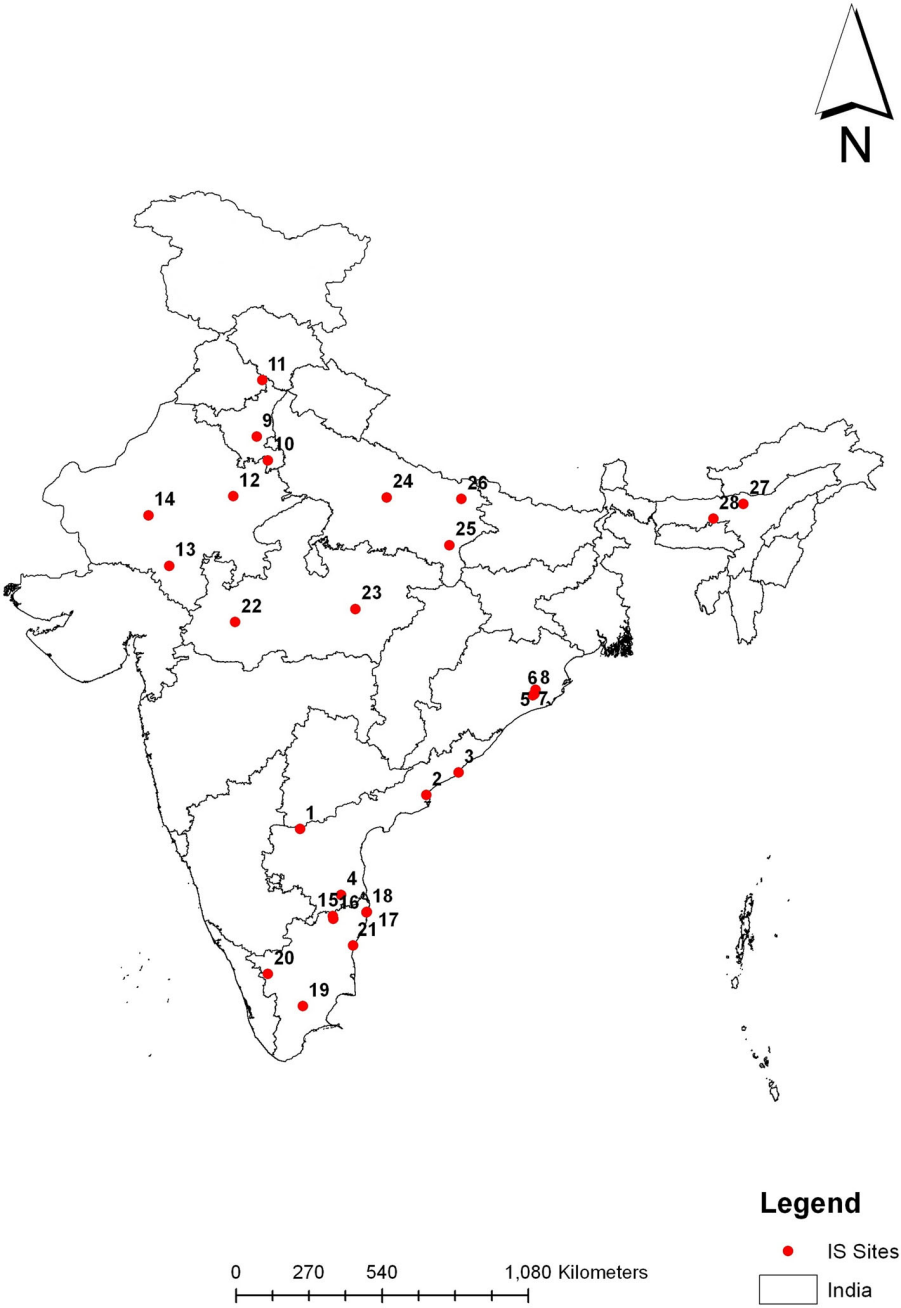
Sentinel hospitals in intussusception surveillance in India. Legends: 1 Kurnool, 2 Kakinada, 3 Vishakhapatnam, 4 Tirupathi, 5 Cuttack, 6 SUM, Bhubaneswar, 7 KIMS, Bhubaneswar, 8 HITECH, Bhubaneswar, 9 Rohtak, 10 Mewat, 11 Chandigarh, 12 Jaipur, 13 Udaipur, 14 Jodhpur, 15 CMC, Vellore 16 GVMC, Vellore, 17 ICH, Chennai, 18 KKCTH, Chennai, 19 Madurai, 20 Coimbatore, 21 Puducherry, 22 Indore, 23 Jabalpur, 24 Lucknow, 25 Varanasi, 26 Gorakhpur, 27 Tezpur, 28 Guwahati. *Source: Community Health and Training Center, Christian Medical College, Vellore*

The multi-centric evaluation for intussusception is at 28 sentinel hospitals in nine states of India for four years (Fig. 1). Based on the recent figures, the sentinel hospitals should be able to admit 40–50 cases per state per year (Additional file 1: Table S1). For the primary objective, the self-controlled case-series methodology will investigate the temporal association between the transient exposure and outcome, in which the individual with the outcome of interest act as his/her own control [22]. The secondary objective of evaluating potential infectious etiologies will be performed through a matched case-control design.

### Subjects

#### Primary objective

All children less than two years of age with intussusception presenting to sentinel hospitals are eligible for recruitment. The inclusion criteria for recruiting cases into surveillance program are; i) age less than 2 years, and ii) meeting level 1 diagnostic certainty for intussusception as per Brighton collaboration criteria. Diagnostic certainty as per level 1 Brighton collaboration criteria are the confirmation of intussusception during surgery and/or by specific radiologic findings (if reduced by pneumatic/hydrostatic/contrast enema) or at autopsy [23].

#### Secondary objective

All children with intussusception enrolled into surveillance for primary objective will serve as cases. For each case identified in the surveillance, a matched control is enrolled. The criteria for matching are; i) age (within ± one month of the case-patient’s age), ii) gender and iii) location (district/region), and iv) control should have a diagnosis unrelated to any gastrointestinal illness and v) control should be from the hospital where the case was enrolled. Each control is to be enrolled within 30 days of the case-patient’s enrollment.

### Sample size

#### Primary objective

Intussusception cases presenting to sentinel hospitals are to be enrolled throughout the surveillance period. To detect a relative incidence of 2, with a 21-day risk period after any dose, with 80% power and 5% level of significance, we require 160 intussusception cases vaccinated with ROTAVAC.

#### Secondary objective

To demonstrate a 10% difference in pathogen prevalence between case-patients and controls with a power of 80% [21], an estimated 140 case-patients and 140 controls are required.

### Surveillance activities

#### Primary objective

Surveillance staff identify intussusception cases admitted to the hospital by surveying pediatric inpatient wards, surgical theatre logs, and admission logs, in close coordination with the hospital pediatric surgeons and radiologists. On identification of a possible case, the surveillance physician ascertains the eligibility and enrols the child. The surveillance staff complete a case report form (CRF) and obtains a copy of ultrasound report along with image, hospital procedure/treatment notes and a copy of the vaccination record.

#### Secondary objective

A control is enrolled for each case by screening the hospital admission logs of paediatrics, pediatric surgery, and pediatric orthopaedics departments. After identifying a potential control, the surveillance physician checks all the criteria before enrollment. Once the physician ascertains the eligibility, the surveillance staff completes a CRF for the control. A stool specimen is collected from all cases and controls.

Written informed consent is obtained from parents/legal guardians of both cases and controls prior to the enrolment. Data collection forms contain unique identifiers to permit identification of participants. At each sentinel hospital, a link between the unique identifier, name of the participant, and laboratory specimen numbers is maintained. This link between name and unique identifier and laboratory specimen will be destroyed after data collection and analysis.

### Specimen collection

For the secondary objective, stool specimens are collected from both cases and controls. A bulk stool specimen (~ 5 ml) is obtained from each enrolled child, preferably on the day of presentation to hospital. In case of any delay, attempts are made to obtain a stool specimen within 48 h of hospital admission to rule out nosocomial infection. The stool specimen is collected in a sterile screw-top container labelled with a unique identification number and date of collection. At the sentinel hospitals, stool specimens are stored at − 20 ^ͦ^ C until shipment to referral laboratory at CMC, Vellore. Once in a month, stool specimens are sent to the referral laboratory in a vaccine carrier with frozen gel packs.

### Laboratory methods

**Fig. 2:**
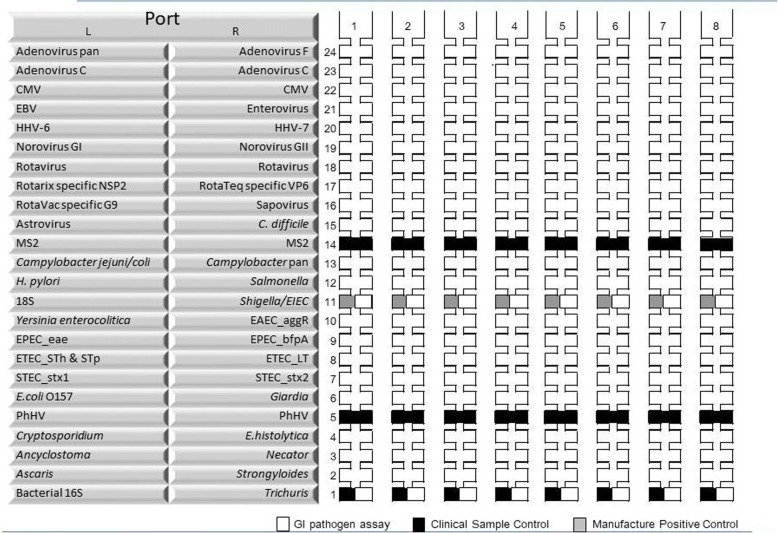
Taqman array card (TAC) for enteropathogen testing

For the secondary objective, testing for the presence of multiple enteropathogens in stool samples from intussusception cases and their matched controls is by using custom made Taqman array card (TAC) assays [24]. Briefly, total nucleic acid is extracted from stool samples and tested for enteropathogen targets including enteric viruses, bacteria and parasites using arrayed singleplex real-time polymerase chain reaction (qPCR) assays. Figure 2 shows all the enteropathogen targets included on these assays for this evaluation.

### Data management

Once a month, completed CRFs of all the children enrolled into surveillance (cases and controls) along with supportive documents and stool specimens are sent to the referral laboratory at CMC, Vellore. On receipt of CRFs and stool samples, an acknowledgement e-mail communication along with any issues found with the quality and quantity of the samples is sent to the sentinel hospitals. At the central data processing centre, all CRFs are stored in a secure, locked cabinet. Trained personnel do data entry into an electronic database using structured query language. Forms with missing data are reviewed and if the program coordinator determines that it is possible to recover the data, the site investigator will attempt to obtain the missing information. Data quality for the surveillance is checked through on-going assessment of sentinel hospital performance.

### Monitoring surveillance

After initiation of surveillance, sentinel sites are visited once in 3 months. At each visit, the sites are evaluated using a monitoring checklist (Additional file 2: Table S2), which recorded performance in terms of enrollment of children with intussusception, collection of ultrasound reports along with images, collection of procedure/treatment notes, enrolling the matched controls, collection of adequate stool samples and obtaining copies of the vaccination cards. Every year, a collaborators’ meeting is organized to discuss the work done by each site and to enable collaborators to provide feedback to individual sites. The hospitals not meeting performance criteria are excluded from subsequent surveillance based on monitoring reports.

### Analysis plan

#### Primary objective

Descriptive analyses of demographic, clinical, and treatment information will be performed for cases identified in the intussusception surveillance. The self-controlled case-series method will be used to assess the intussusception risk after ROTAVAC administration [25, 26]. The relative incidence of intussusception during the risk periods of 1–7 days, 8–21 days and 1–21 days post ROTAVAC vaccination for dose 1, 2 and 3 will be estimated.

#### Secondary objective

Matched case-control analysis for intussusception etiologies will include comparison of demographic information, preceding clinical symptoms, and feeding patterns between case-patients and controls using the chi-square or Fisher’s exact tests. Conditional logistic regression will be used to estimate adjusted population attributable fraction of intussusception due to different pathogens. Attributable cases will be calculated for each pathogen [27, 28].

## Discussion

### Challenges in surveillance

Initial reluctance in participation was overcome by site investigators’ meetings before initiation, which helped to explain the project design, surveillance activities and significance of the project. During the surveillance, determining the vaccination status of the child and obtaining a photocopy of the vaccine card is challenging, as parents do not always carry the vaccine cards. In such cases, parents are asked to send a copy of the vaccination card by post or by email. In certain cases, the surveillance staff travels to the child’s home to obtain copies. For subjects with no immunization cards available, the surveillance staff contact the auxiliary nurse midwife at health sub-centers [29] who maintain government immunization records to verify receipt of rotavirus vaccine. For children vaccinated at private hospitals, attempts are made to identify the manufacturer by contacting the health facility where the child received the rotavirus vaccine. For the etiology evaluation, enrolling controls satisfying all the matching criteria was challenging, especially at big referral hospitals having cases from two or more states. Surveillance physicians had to carefully scrutinize all the potential controls before enrolling a matched control.

Monitoring and demonstrating the safety of the vaccine in terms of intussusception after introduction of the vaccine into the routine immunization programme is important to ensure continued support and commitment for the rotavirus vaccination program. Anticipated public health benefits from this surveillance include the generation of information useful to public health officials and to national governments in making decisions through future risk-benefit analysis of the vaccine. This evaluation will also generate evidence for other low- and middle- income countries (LMIC) planning to introduce rotavirus vaccine.

The knowledge on the etiology of intussusception is limited worldwide [30]. Evaluating the infectious agents present in stool samples from intussusception cases and matched controls may help to determine potential infectious etiologies of intussusception, and will assist in further understanding the association, if any, between rotavirus vaccination and intussusception.

## Additional files

Additional file 1: **Table S1.** Baseline data from sentinel hospitals included in the intussusception surveillance. (DOCX 15 kb)

Additional file 2: **Table S2.** Monitoring checklist for sentinel hospitals in intussusception surveillance. (DOCX 26 kb)

## Abbreviations

CDC: Centers for Disease Control and Prevention
CMC: Christian Medical College
CRF: Case report form
ICMR: Indian Council of Medical Research
LMIC: Low- and middle- income countries
MoU: Memorandum of Understanding
RV: Rotavirus
TAC: Taqman Array Card
THSTI: Translational Health Science and Technology Institute (THSTI)
UIP: Universal Immunization Programme
WHO: World Health Organization
