# Supplementary material for: Safety monitoring of ROTAVAC vaccine and etiological investigation of intussusception in India: study protocol
Source: BMC Public Health. 2018 Jul 20;18:898. doi: 10.1186/s12889-018-5809-7 (PMC6053826; doi:10.1186/s12889-018-5809-7)
Supplement: Supplementary file 1 — Table S1. Baseline data from sentinel hospitals included in the intussusception surveillance. (DOCX 15 kb) [file 12889_2018_5809_MOESM1_ESM.docx]

Table S1- Baseline data from sentinel hospitals included in the intussusception surveillance

| **Sl. no.** | **Surveillance Network Centers** | **No. of under two intussusception admissions a year*** |
| --- | --- | --- |
| 1 | Kurnool Medical College, Kurnool, Andhra Pradesh | 8 |
| 2 | Government General hospital, Kakinada, Andhra Pradesh | 30 |
| 3 | King George Hospital, Vishakhapatnam, Andhra Pradesh | 10 |
| 4 | Sri Venkateswara Medical College, Tirupati, AP | 8 |
| 5 | Sardar Vallabhai Patel Post Graduate Institute of Paediatrics, Cuttack, Odisha | 34 |
| 6 | Kalinga Institute of Medical Sciences, Bhubaneswar, Odisha | 5 |
| 7 | Institute of Medical Sciences and SUM Hospital, Bhubaneswar, Odisha | 10 |
| 8 | Hi-Tech Hospital, Bhubaneswar, Odisha | 2 |
| 9 | Pandit Bhagwat Dayal Sharma Post Graduate Institute of Medical Sciences, Rohtak, Haryana | 10 |
| 10 | Shaheed Hasan Khan Mewati Government Medical College, Mewat, Haryana | 5 |
| 11 | Post Graduate Institute of Medical Education and Research, Chandigarh | 35 |
| 12 | Savai Man Singh Medical College, Jaipur, Rajasthan | 106 |
| 13 | Rabindranath Tagore Medical college, Udaipur, Rajasthan | 24 |
| 14 | Dr. Sampurnanand Medical College, Jodhpur, Rajasthan | 50 |
| 15 | Christian Medical College, Vellore, Tamil Nadu | 36 |
| 16 | Government Vellore Medical College, Tamil Nadu | 8 |
| 17 | The Institute of Child Health and Hospital for Children, Chennai, Tamil Nadu | 61 |
| 18 | Kanchi Kama Koti Child Trust Hospital, Chennai, Tamil Nadu | 44 |
| 19 | Government Medical College, Madurai, Tamil Nadu | 47 |
| 20 | Government Medical College, Coimbatore, Tamil Nadu | 16 |
| 21 | Jawaharlal Nehru Institute of Post-graduate Medical Education & Research (JIPMER), Puducherry | 34 |
| 22 | Mahatma Gandhi Memorial Medical college, Indore, Madhya Pradesh | 30 |
| 23 | NSCB Medical college, Jabalpur | 12 |
| 24 | King George Medical College, Lucknow, Uttar Pradesh | 19 |
| 25 | Institute of Medical Sciences, Banaras Hindu University, Varanasi, Uttar Pradesh | 12 |
| 26 | BRD Medical College, Gorakhpur, Uttar pradesh | 10 |
| 27 | Baptist Christian Hospital, Tezpur, Assam | 4 |
| 28 | Government Medical college, Guwahati, Assam | 14 |

*Retrospective data for the year 2015
