# Supplementary material for: Safety monitoring of ROTAVAC vaccine and etiological investigation of intussusception in India: study protocol
Source: BMC Public Health. 2018 Jul 20;18:898. doi: 10.1186/s12889-018-5809-7 (PMC6053826; doi:10.1186/s12889-018-5809-7)
Supplement: Supplementary file 2 — Table S2. Monitoring checklist for sentinel hospitals in intussusception surveillance. (DOCX 26 kb) [file 12889_2018_5809_MOESM2_ESM.docx]

Table S2 Monitoring checklist for sentinel hospitals in intussusception surveillance

| **Date of Review:** |  | |
| --- | --- | --- |
| **State:** | | |
| **Institute*:*** | | |
| Address: | | |
| **Name of PI:**  **Email:** | | **Name of CO-PI:**  **Email:** |
| **Reviewer(s):** | | |

**Part I: Performance in recruitment**

| 1. **1** | **Recording of recruitment** |
| --- | --- |
| - 1. 1.1 | Date of surveillance initiation |
| - 1. 1.2 | Number of children admitted with intussusception (from hospital logbook for surveillance) |
| - 1. 1.3 | Is there a source from which the details in the hospital logbook can be checked:  If yes, describe the source below (e.g. admission records, ward book, electronic medical records) |
| - 1. 1.4 | How many locations are children recruited from? (e.g. Pediatric surgery wards, emergency room, Radiology department)  Please list below all locations from which children are recruited at this site: |
| - 1. 1.5 | Number of intussusception cases enrolled into surveillance so far (Informed consent form completed) |
| - 1. 1.6 | Are reasons for non-enrollment recorded? If yes, where? |
| - 1. 1.7 | Number of intussusception cases for whom case report form was filled so far |
| - 1. 1.8 | Are reasons for non-completion of CRF recorded? If yes, where? |
| - 1. 1.9 | Number of intussusception cases for whom stool sample was collected so far |
| 1.10 | Are reasons for non-collection of stool sample recorded? If yes, where? |
| 1.11 | Number of intussusception cases for whom ultrasound report was collected so far |
| 1.12 | Reasons for non-collection of ultrasound report |
| 1.13 | Number of intussusception cases for whom ultrasound image was collected so far |
| 1.14 | Reasons for non-collection of ultrasound images |
| 1.15 | Number of intussusception cases for whom procedure/treatment notes was collected so far |
| 1.16 | Reasons for non-collection of procedure/treatment notes |
| 1.17 | Number of control children enrolled into surveillance (Informed consent form completed) |
| 1.18 | Number of control children with completed CRF |
| 1.19 | Reasons for non-completion of CRF |
| 1.20 | Number of controls for whom stool sample was collected |
| 1.21 | Reasons for non-collection of stool sample |
| 1.22 | Problems encountered by the site in enrolling controls |
| 1. **2** | **Review of informed consent forms (ICF)** |
| - 1. 2.1 | Number of ICFs reviewed |
| - 1. 2.2 | Are the forms filed in a location where they are easily accessible? |
| - 1. 2.3 | Can the forms be checked against the recruitment information (section 1)? |
| - 1. 2.4 | Are all the forms available? (at each visit check forms since previous visit)  If no, record project ID numbers of missing ICFs: |
| - 1. 2.5 | Are the forms legible? If no, record the number of forms with incomplete/unclear information |
| - 1. 2.6 | Are the signatures of parents/guardians clear? |
| - 1. 2.7 | Are the forms signed by the investigator? |
| - 1. 2.8 | What are the problems encountered by the site in completing this information? |
| 1. **3** | **Completion of Case Report Forms** |
| - 1. 3.1 | Number of CRFs reviewed: |
| - 1. 3.2 | Number of CRFs with missing data fields:  Record below which fields have missing data |
| - 1. 3.3 | Is there a signature and a date for completion of CRF: |
| - 1. 3.4 | Is there a record of stool sample collection? |
| - 1. 3.5 | Can the stool sample collection be verified against the hospital logbook? |
| - 1. 3.6 | Can the stool sample collection be verified against the sample transmittal log? |
| - 1. 3.7 | What are the problems encountered by the site in completing this information? |
| **4** | **Collection of vaccination information** |
| 4.1 | What are the sources of vaccination information in the CRFs examined in Section 3? List below: |
| - 1. 4.2 | Can dates be verified on the CRFs from the source? |
| - 1. 4.3 | What are the problems encountered by the site in completing this information? |
| **5.** | **Collection of stool samples** |
| 5.1 | Number of samples collected so far: |
| 5.2 | Number of samples checked on this visit |
| 5.3 | Is the information on the containers complete? If no, how many samples are incorrectly labelled? |
| 5.4 | Are the samples stored appropriately?  State below location of storage: |
| 5.5 | Are the samples checked on this visit adequate volume?  If no, how many were inadequate? |
| 5.6 | Can the stool samples be verified against the hospital logbook? |
| 5.7 | Can the stool samples be verified against the CRF? |
| 5.8 | Can the stool sample collection be verified against the sample transmittal log? |
| 5.9 | What are the problems encountered by the site in completing this information? |

**Part II: Site Profile**

| 1. **1** | **Staff** |
| --- | --- |
|  | Number of scientific and technical staff assigned to the surveillance (include post-graduates etc) |

| **Name of staff** | **Position Title or Duties** | **Full-time or Part-time for intussusception responsibilities** | **% of time spent working on Intussusception**  **surveillance** | **Years of experience in Site** |
| --- | --- | --- | --- | --- |
|  |  |  |  |  |
|  |  |  |  |  |
|  |  |  |  |  |
|  |  |  |  |  |
|  |  |  |  |  |

| 2. | **Space and equipment** |
| --- | --- |
| 2.1 | Where records are stored (ICF/CRF etc)? |
| 2.2 | Is there a refrigerator for storage of stool samples?  State location |

| 3. | **Management and supervision** |
| --- | --- |
| 3.1 | The lines of supervision and accountability are clear to all staff |
| 3.2 | Written protocols are available and arrangements are in place for periodic review and evaluation |
| 3.3 | Arrangements are made for back-up staff to perform work during staff absences (e.g. vacation, sick leave, etc) |

**Part III: Summary of Review**

**Indicators:**

| **1.** | Enrollment of >90% of admitted cases of intussusception |
| --- | --- |
| **2.** | Collection of adequate stools from >90% of intussusception cases |
| **3.** | Complete documentation (ICF/CRF) in >90% of enrolled intussusception cases |
| **4.** | Collection of vaccination record (patient retained/government) for >90% of enrolled cases |
| **5.** | Collection of procedure/treatment notes for >90% of enrolled cases |
| **6.** | Enrollment of >90% of matched controls |
| **7.** | Collection of adequate stools from >90% of enrolled controls |
| **8.** | Complete documentation (ICF/CRF) in >90% of enrolled controls |
| *SUMMARY, COMMENTS AND RECOMMENDATIONS****:*** | |
